# Evaluation of Patient Reported Safety and Efficacy of Cannabis From a Survey of Medical Cannabis Patients in Canada

**DOI:** 10.3389/fpubh.2021.626853

**Published:** 2021-05-20

**Authors:** Shaina P. Cahill, Stephanie E. Lunn, Patrick Diaz, Jonathan E. Page

**Affiliations:** ^1^Aurora Cannabis Inc., Edmonton, AB, Canada; ^2^Department of Botany, University of British Columbia, Vancouver, BC, Canada

**Keywords:** cannabidiol, tetrahydrocannabinol, quality of life, sleep disorder, post-traumatic stress disorder, pain, medical cannabis, real-world data

## Abstract

With the medical use of cannabis permitted in Canada since 2001, patients seek to use this botanical drug to treat a range of medical conditions. However, many healthcare practitioners express the need for further scientific evidence around the use of medical cannabis. This real-world evidence study aimed to address the paucity of scientific data by surveying newly registered medical cannabis patients, before beginning medical cannabis treatment, and at one follow up 6 weeks after beginning medical cannabis treatment. The goal was to collect data on efficacy, safety and cannabis product type information to capture the potential impact medical cannabis had on patient-reported quality of life (QOL) and several medical conditions over a 6-week period using validated questionnaires. The 214 participants were mainly male (58%) and 57% of the population was older than 50. The most frequently reported medical conditions were recurrent pain, post-traumatic stress disorder (PTSD), anxiety, sleep disorders [including restless leg syndrome (RLS)], and arthritis and other rheumatic disorders. Here we report that over 60% of our medical cannabis cohort self-reported improvements in their medical conditions. With the use of validated surveys, we found significant improvements in recurrent pain, PTSD, and sleep disorders after 6 weeks of medical cannabis treatment. Our findings from patients who reported arthritis and other rheumatic disorders are complex, showing improvements in pain and global activity sub-scores, but not overall changes in validated survey scores. We also report that patients who stated anxiety as their main medical condition did not experience significant changes in their anxiety after 6 weeks of cannabis treatment, though there were QOL improvements. While these results show that patients find cannabis treatment effective for a broad range of medical conditions, cannabis was not a remedy for all the conditions investigated. Thus, there is a need for future clinical research to support the findings we have reported. Additionally, while real-world evidence has not historically been utilized by regulatory bodies, we suggest changes in public policy surrounding cannabis should occur to reflect patient reported efficacy of cannabis from real-world studies due to the uniqueness of medical cannabis's path to legalization.

## Introduction

Cannabis has been used for centuries to treat ailments in humans (1), with the earliest peer-reviewed clinical studies reported in 1843 (2). Although there is a long history of anecdotal evidence supporting the potential therapeutic benefits of cannabis (3), political, legal and social influences have significantly hindered research efforts. Thus, while medical cannabis is authorized for use in Canada, medical guidance documents produced by Canadian physicians' colleges continue to highlight the lack of efficacy data for cannabis in a diverse number of medical conditions (4–7). To date, physicians most often advocate for medical cannabis use in cases where all other conventional options have been exhausted.

The available scientific data on the efficacy of cannabinoid-based therapies spans a wide range of medical conditions that include mental health conditions, nausea and vomiting, chronic pain, epilepsy, and sleep issues (8). Additionally, different cannabinoid-based therapies (both in terms of routes of administration and cannabinoid ratios) have been utilized in these various scientific studies. Cannabis contains over 100 cannabinoids (phytocannabinoids) and up to 200 terpenes, with the cultivar's genetic makeup and specific environmental conditions influencing the ratios and levels of these compounds found in the mature plant (9, 10). Δ^9^-tetrahydrocannabinol (THC) and cannabidiol (CBD) are thought to be responsible for the majority of the physiological effects induced by cannabis (8). THC and its metabolite, 11-hydroxy tetrahydrocannabinol (11-OH-THC), are primarily responsible for causing intoxication (11–13). In contrast to THC, CBD is non-intoxicating at medically relevant doses (8). While each of these cannabinoids have been tied to specific physiological effects on their own, some authors have proposed that cannabinoids, including minor cannabinoids other than THC and CBD, and/or terpenes work together synergistically, in what has been termed the “entourage effect”(14). Therefore, different ratios of THC:CBD may have different therapeutic effects. This makes the interpretation and application of the currently available scientific data on the efficacy of medical cannabis for a specific medical condition complex and unlike standard pharmaceuticals, where a single drug compound is approved for one medical condition.

This study aimed to address the significant knowledge gap for healthcare practitioners seeking to treat patients with cannabis by surveying medical cannabis patients when they first became patients of a Canadian licensed producer, before beginning medical cannabis treatment, and at one follow up time point 6 weeks after beginning medical cannabis treatment. These surveys aimed to capture the potential impact medical cannabis had on patient-reported quality of life (QOL) and medical conditions over a 6 week period by using multiple validated questionnaire conditions for: QOL, recurrent pain, anxiety, arthritis and other rheumatic disorders, post-traumatic stress disorder (PTSD) and sleep disorders, including restless leg syndrome (RLS). This study particularly focused on investigating what types of pain patients reported experiencing and if they found medical cannabis effective in treating their pain. This examination line arose from previous real-world evidence reported by Ueberall et al. that 12-weeks of nabiximols treatment significantly improved pain for patients with neuropathic chronic pain and mixed pain but was not effective and/or worsened pain symptoms for patients with nociceptive pain (15).

By surveying medical cannabis patients who had access to numerous THC:CBD ratios and asking which they felt was most effective at treating their specific medical conditions, this study aimed to gather real-world evidence that may inform future clinical research as well as healthcare practitioner practices around authorizing medical cannabis.

## Methods

Administration of the survey was through e-mail to newly registered patients with the Canadian licensed producer, MedReleaf^®^, a wholly owned subsidiary of Aurora Cannabis Inc^®^ between January through August 2020. Newly registered patients received an invitation via e-mail to complete a voluntary online intake survey after their first medical cannabis order. After clicking the link, respondents were taken to the Qualtrics survey site to complete the survey. Six weeks following completion of the initial intake survey, patients were invited to complete a follow-up survey. Patients provided consent for the collection and use of their data, and no incentives were provided to either complete the survey or select any particular cannabis product.

Patients were included in this analysis if they completed the intake and follow-up surveys between January 2020 through the end of August 2020. Data from patients who did not complete both intake and follow-up surveys during the January through August 2020 period were not included. Baseline survey data from the intake survey were filtered to include only patients who completed the 6-week follow-up survey for analysis. Patients who selected “other” to answer any question were asked to specify, and any patient who provided only one answer was included in the analysis while responses including more than one answer were not included. Data was summarized with statistical parameters of median, mean, range, standard deviation (SD), and percentage of the total, where appropriate. All comparisons were assessed by either paired *t*-tests or Wilcoxon test. The Shapiro-Wilk test of normality was used to identify non-normal distribution. In all cases, significance was set at *P* < 0.05, with a trend defined as *P* = 0.1 <0.05. All statistical analyses and graphs were performed using Prism Version 8.

### Survey Design

The survey was designed using scientific literature and guidance from health care professionals with experience using medical cannabis for patient care. The survey was presented in a dynamic format customized to individual responses, where responses determined the subsequent questions asked (e.g., if a patient did not report pain, no further pain-related questions were asked). Patients were also given the option to skip questions they did not want to answer. For specific questions, patients were given the option to select “prefer not to answer” or “other” and were provided with a text box to provide their own response. As such, each patient completing the survey answered a unique set of questions, and each question in the survey received different numbers of responses.

Data was collected *via* self-completed web-based surveys conducted from January 2020 through the end of August 2020. Demographics (including age, sex, ethnicity, and employment status) and medical history information was collected from patients during both the intake and follow-up surveys. Patients were also asked if they experienced recurring pain and to identify the main medical condition for which they were seeking medical cannabis treatment.

During the follow-up survey, patients were asked to report any changes in their main medical condition or recurrent pain, which cannabis products they perceived the changes could be attributed to, and how long patients found it took medical cannabis to affect their overall condition. After being asked about any changes in their medical condition or recurrent pain, patients were provided a drop-down menu to choose which cannabis product they attributed these changes to, with product options including dried herbal cannabis, cannabis oils, cannabis softgels and cannabis vaporizers. Given the extensive product catalog, the resulting data was categorized based on cannabinoid content into previously described chemotypes (16), where high THC is defined as a product containing >15% THC, high CBD is a product that contains CBD as the principle cannabinoid while consisting of <1% THC, and a balanced product has a near equal amount of THC to CBD. Additionally, patients reported any side effects they attributed to the 6-weeks of medical cannabis treatment.

### Validated Assessment Tools

To assess changes in reported medical conditions and QOL measures, both validated questionnaires and self-reported changes in health outcomes were utilized.

#### EuroQOL 5-Dimensions 5-Levels

To assess patients' QOL, all patients completed the EuroQol-5D-5L (EQ-5D-5L), a two-part tool consisting of the EQ-5D-5L descriptive system and the EQ Visual Analog Scale (EQ-VAS) (17, 18). The descriptive system measures five domains (mobility, self-care, usual activities, pain or discomfort, anxiety or depression), each with five levels of severity: no problems, slight problems, moderate problems, severe problems, and extreme problems. For the descriptive system section of the EQ-5D-5L, several country-specific preference-based scoring systems have been developed. For this study, index scores based on societal preferences in Canada to represent EQ-5D index scores were used (18) (see Supplementary Figure 1). The EQ-VAS is a 20 centimeter (cm) vertical scale from 0 to 100 whereby the upper endpoint of the scale corresponds to the “best health you can imagine,” and the lower corresponds to the “worst health you can imagine.”

#### Pain Outcomes Questionnaire—Short Form

The POQ-SF was administered to all patients who indicated that they experience recurring pain. The POQ-SF is adapted from the longer POQ-VA questionnaire (19). The POQ-SF is a 19-item inventory with primary pain items rated on a 11-point (0-10) Likert-type scale. The tool yields an overall score, a pain numeric rating scale (NRS) and five subscales measuring activities of daily living (ADL), negative affect (NA), mobility, vitality and fear.

#### Depression, Anxiety, and Stress Scale

The DASS-21 was administered to any patient who indicated their main medical condition was anxiety or depression. The DASS-21 is a 21-item questionnaire with three 7-item subscales: Depression, Anxiety and Stress (20, 21). Items consist of statements referring to the past week, and each item is scored on a 4-point scale (0 = “Did not apply to me at all,” to 3 = “Applied to me very much, or most of the time”). The DASS-21 is derived from DASS-42, which is a 42-item measure of the same three constructs.

#### Patient Activity Scale-II

The PAS-II was administered to any patient who indicated their main medical condition was arthritis or another rheumatic disorder. The PAS-II is a modified version of the PAS-I which is recommended as a disease activity measure by the American College of Rheumatology (22). The PAS-II consists of the HAQ-II and a 10 cm visual analog scale (VAS) for both pain and patient global assessment (23).

#### Short PTSD Rating Interview

The SPRINT was administered to any patient who indicated their main medical condition was PTSD. The SPRINT consists of four items corresponding to each of the four PTSD symptom clusters (intrusion, avoidance, numbing and hyperarousal), as well as four additional questions assessing, respectively, somatic distress, being upset by stressful events, interference with work or daily activities and relationships among family or friends (24). Each item is rated on a 5-point Likert scale from not at all (0) to very much (4). The SPRINT also contains two additional items to measure global improvement according to percentage change and severity rating, administered only during the follow-up survey (24).

#### Pittsburgh Sleep Quality Index

The PSQI was administered to any patient who indicated their main medical condition as a sleep disorder, which included RLS. The PSQI assesses sleep quality during the previous month and contains 19 self-rated questions yielding seven components; subjective sleep quality, sleep latency, sleep duration, habitual sleep efficiency, sleep disturbances, use of sleep medication and daytime dysfunction (25). Each item is rated on a 4-point Likert scale from “not during the past month” (0) to “three or more times a week” (3), yielding a global PSQI score between 0 and 21(25).

## Results

### Demographics

Of the 608 patients who completed the intake survey, 214 went on to complete the 6-week follow-up survey. Patients' age ranged from 19–79 years (50.7 ± 11.2 years; 58% male), with 57% of the cohort over the age of 50 years. Eighty-six percent of the population reported their ethnicity to be White/Caucasian, and 40 and 34% of patients reported their employment status to be employed and retired, respectively. Forty-seven percent of patients reported being a veteran, where a veteran was defined as a past member of the Canadian Armed Forces, Royal Canadian Mounted Police, Corrections Services Canada, Canadian Coast Guard, Paramedic or Peace Officer (Table 1).

**Table 1:** Demographic information of the medical cannabis patient cohort.

**Demographic information**	***n* (%)**
Age	(Mean age: 50.7 years)
0–19	2 (0.9)
20–29	3 (1.4)
30–39	33 (15.4)
40–49	54 (25.2)
50–59	75 (35.0)
60–69	40 (18.7)
70–79	7 (3.3)
**Biological sex**	
Female	71 (33.2)
Male	124 (57.9)
Other	19 (8.9)
**Ethnicity**	
Asian	4 (1.9)
Black/Black Canadian	2 (09)
Hispanic/Latino	0 (0.0)
Indigenous Canadian	14 (6.5)
White/Caucasian	185 (86.4)
Other	7 (3.3)
Prefer not to answer	2 (0.9)
**Employment**	
Employed (Full- and Part- Time)	85 (39.7)
Homemaker	8 (3.7)
Not employed	14 (6.5)
Retired	72 (33.6)
Self-employed	14 (6.5)
Student	6 (2.8)
Prefer not to answer	15 (7.0)
**Veteran status**	
No	114 (53.3)
Yes	100 (46.7)
**Previous cannabis experience**	
No	62 (29.0)
Yes	145 (67.8)
Prefer not to answer	7 (3.3)

Patients were also asked about their previous experience with cannabis. At the time of the intake survey, before patients had begun their medical cannabis treatment with the licensed producer, 68% reported having previous experience with cannabis (Table 1).

### Recurrent Pain

Patients were asked, “Do you experience recurring pain?” If patients reported having recurring pain, they were asked to select “What type of recurring pain do you experience?” from a list of six pain type options. Eighty-six percent of patients reported having recurring pain. Nociceptive (31%) and neuropathic (22%) pain were the most common types of pain reported by these patients (Table 2). Patients also reported the number of years they experienced recurring pain, with 38% of patients reporting experiencing pain between 11–20 years (14.3 ± 10.6, Table 2).

**Table 2:** Information related to recurring pain for the medical cannabis patient cohort.

**Recurring pain**	***n* (%)**
**Experience recurring pain**	
No	31 (14.5)
Yes	183 (85.5)
**Type of recurring pain**	
Neuropathic	40 (21.9)
Nociceptive	57 (31.1)
Phantom	2 (1.1)
Psychogenic	35 (19.1)
Not sure	16 (8.7)
Other	33 (18.0)
**Years with recurring pain**	(Mean number of years: 14.6 years)
1–5	33 (18.0)
6–10	48 (26.2)
11–20	69 (37.7)
20+	33 (18.0)

**Figure 1:**
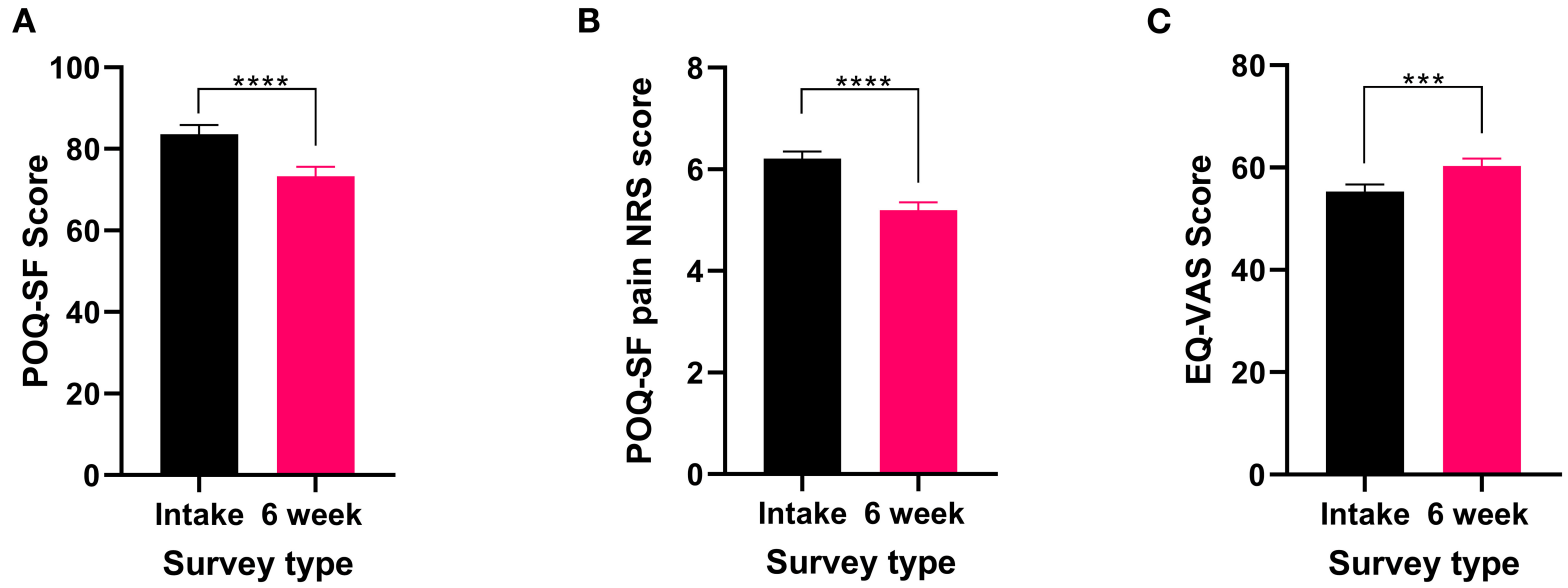
Validated survey scores from the intake survey (baseline) and the 6-week follow-up survey for patients reporting recurrent pain. Overall POQ-SF, pain NRS score and the five subscales of the POQ-SF (see Supplementary Figure 2), showed improvements after 6 weeks of medical cannabis treatment. **(A)** Overall POQ-SF score significantly decreased after 6 weeks of medical cannabis treatment (*t*_182_ = 6.74 *P* < 0.0001). **(B)** The pain NRS score reduced significantly (Wilcoxon test *P* < 0.001) from 6.2 ± 1.8/10 at baseline to 5.2 ± 2.1/10 at the 6-week follow-up. **(C)** EQ-VAS score significantly improved from the intake survey to the 6-week follow-up survey (*t*_182_ = 3.53 *P* < 0.001), (****P < 0.0001, ***P < 0.001).

In addition to reporting the presence of recurring pain, all patients who reported experiencing recurrent pain completed the POQ-SF and the EQL-5D-5L. These validated surveys were used to capture current pain, the effects of pain on QOL and any improvements from the six weeks of cannabis treatment. Both the POQ-SF total score (*t*_182_ = 6.74 *P* < 0.0001) and the pain NRS (Wilcoxon test *P* < 0.0001) significantly improved after 6 weeks of medical cannabis treatment (Figures 1A,B). For QOL, EQ-VAS score significantly improved (*t*_182_ = 3.53 *P* < 0.001, Figure 1C) from the intake survey to the 6-week follow-up survey. Thus, patients who reported they had recurrent pain experienced an improvement in their pain and overall QOL after 6 weeks of medical cannabis treatment.

**Figure 2:**
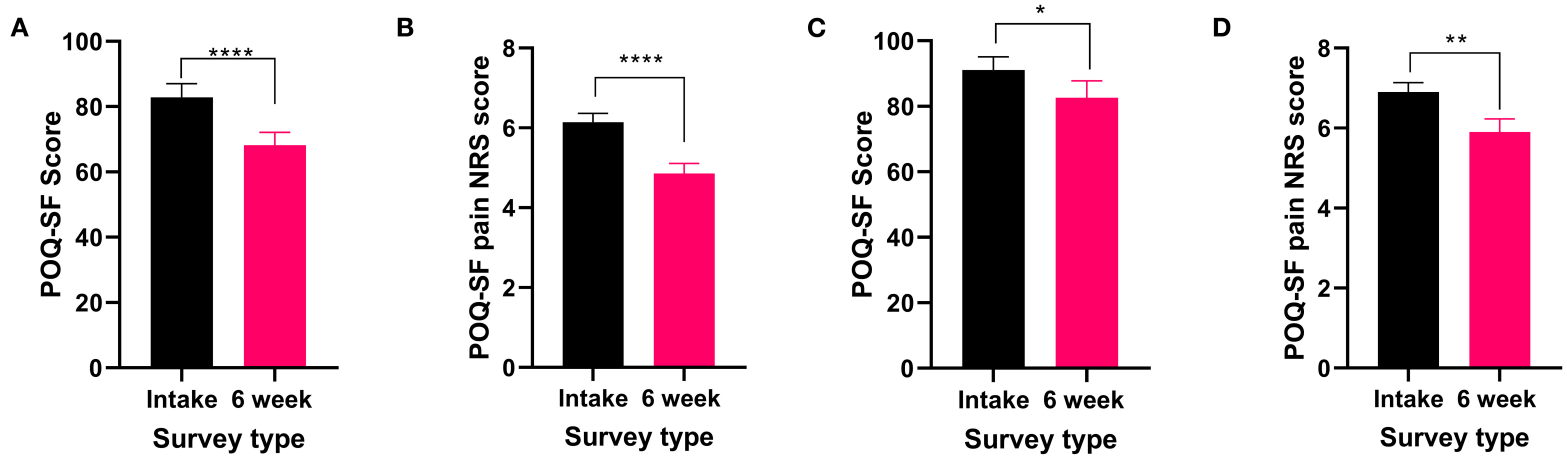
Validated survey scores from the intake survey (baseline) and 6-week follow-up survey for patients reporting nociceptive and neuropathic pain types. Overall POQ-SF and the pain NRS scores showed improvements following 6 weeks of medical cannabis treatment. **(A,B)**. Nociceptive pain: **(A)** Overall POQ-SF score significantly decreased after 6 weeks of medical cannabis treatment (*t*_56_ = 5.116 *P* < 0.0001). **(B)** The pain NRS score significantly improved (*t*_56_ = 4.637 *P* < 0.0001) from 6.1 ± 1.7/10 at baseline to 4.9 ± 1.9/10 at the 6-week follow-up. (**C,D)**. Neuropathic pain: **(C)** Overall POQ-SF score significantly decreased after 6 weeks of medical cannabis treatment (*t*_39_ = 2.261 *P* < 0.05). **(D)** The pain NRS score significantly improved (*t*_39_ = 2.994 *P* < 0.01) from 6.9 ± 1.5 /10 at baseline to 5.9 ± 2.1/10 at the 6-week follow-up, (****P < 0.0001, **P < 0.01, *P < 0.05).

The two most commonly reported pain types, nociceptive and neuropathic, both showed significant improvements in the POQ-SF total score (nociceptive: *t*_56_ = 5.116 *P* < 0.0001 Figure 2A and neuropathic: *t*_39_ = 2.261 *P* < 0.05, Figure 2C) and the pain NRS (nociceptive: *t*_56_ = 4.637 *P* < 0.0001 Figure 2B and neuropathic: *t*_39_ = 2.994 *P* < 0.01, Figure 2D) following 6 weeks of medical cannabis treatment.

Finally, in the 6-week follow-up survey, patients were asked to indicate which cannabis product (if any) had been the most helpful in treating their recurrent pain. Of those who rated cannabis as being helpful, 33% selected high CBD products and 31% selected high THC products (Table 3 and Supplementary Table 1).

**Table 3:** Information on preferred products for the medical cannabis patients who reported recurrent pain.

**Cannabis Product**	***n* (%)**
	(number of respondents: 173)
High THC	53 (30.6)
High CBD	57 (32.9)
Balanced (THC:CBD)	20 (11.6)
Other	31 (17.9)
None	12 (6.9)

### Main Medical Condition

Patients were asked, “What is the main health condition for which you're seeking treatment with medical cannabis?” a list of 25 medical condition options was provided, and patients could select only one medical condition or provide a different medical condition not listed in a free-text box. The four most frequently selected medical conditions were PTSD (15%), anxiety (15%), arthritis and other rheumatic disorders (13%) and sleep disorders (including RLS) (13%) (Table 4).

**Table 4:** The number (and percentages) of medical cannabis patients who self-reported each medical condition.

**Medical condition**	***n* (%)**
Anxiety	33 (15.4)
Post-traumatic stress disorder (PTSD)	32 (15.0)
Arthritis and other rheumatic disorders	28 (13.1)
Sleep disorder	27 (12.6)
Degenerative disc disorder	22 (10.3)
Other	20 (9.3)
Depression	10 (4.7)
Pain	8 (3.7)
Spinal disc herniation	7 (3.3)
Fibromyalgia	6 (2.8)
Cancer	4 (1.9)
Headaches	3 (1.4)
Migraines	3 (1.4)
Tinnitus	3 (1.4)
Attention deficit hyperactivity disorder (ADHD)	2 (0.9)
Epilepsy	2 (0.9)
Hypertension	2 (0.9)
Irritable bowel syndrome	2 (0.9)
**Years with medical condition**	
<1 year	6 (2.8)
1	3 (1.4)
2	9 (4.2)
3	10 (4.7)
4	8 (3.7)
5	12 (5.6)
6	6 (2.8)
7	1 (0.5)
8	9 (4.2)
9	4 (1.9)
10	7 (3.3)
More than 10 years	139 (65.0)

Patients were also asked to report how many years they had suffered from their identified medical condition from a drop-down list (the maximum option was “more than 10 years”). Overall, 65% of patients reported having their specific medical condition for more than 10 years (Table 4).

Finally, patients were asked during the 6-week follow-up survey about any changes in activity limitations, symptoms, emotions and overall QOL since beginning medical cannabis treatment. Thirty-six percent of patients described that they felt better overall, with definite improvements that had made real and worthwhile differences (Table 5). When asked about how long patients found it took medical cannabis to affect their overall condition, 26% reported the effects of medical cannabis on their condition were experienced immediately (Table 5).

**Table 5:** The number (and percentages) of the self-reported changes from patients after 6 weeks of medical cannabis treatment.

**Self-reported changes**	***n* (%)**
**Overall change in general well-being**	
No change	10 (4.7)
Almost the same	14 (6.5)
A little better	18 (8.4)
Somewhat better	26 (12.1)
Moderately better	56 (26.2)
Better	77 (36.0)
A great deal better	13 (6.1)
**Time for cannabis to produce effects**	
No change	9 (4.2)
Immediately	57 (26.6)
~1 week	50 (23.4)
~2 weeks	46 (21.5)
~3 weeks	23 (10.7)
Over 1 month	29 (13.6)

#### Anxiety

**Figure 3:**
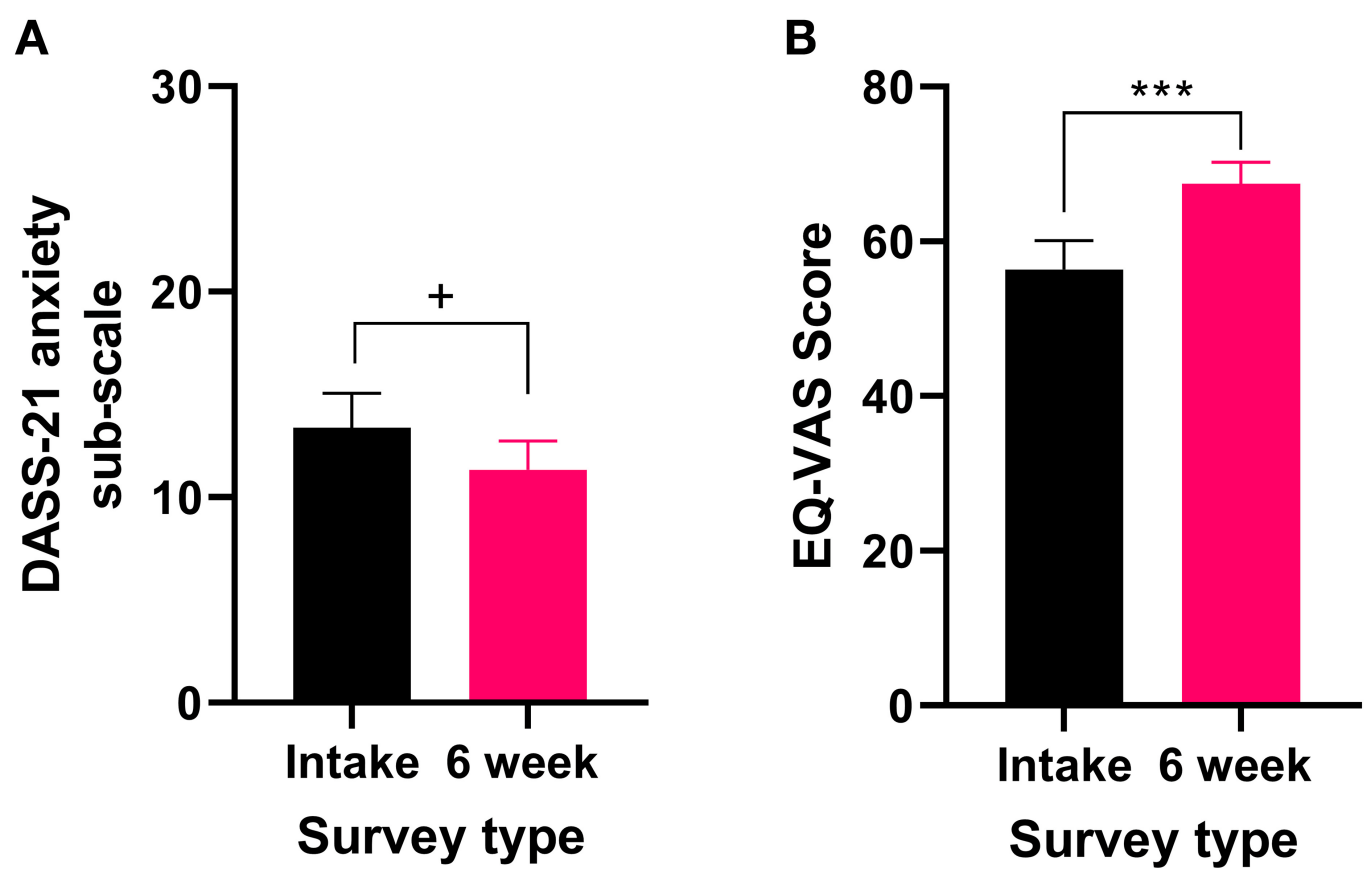
Validated survey scores from the intake survey (baseline) and 6-week follow-up survey for patients reporting anxiety as their primary medical condition. There was an improvement in QOL after 6 weeks of medical cannabis treatment. **(A)** DASS-21 anxiety sub-scale scores were not significantly altered after 6 weeks of medical cannabis treatment (*t*_32_ = 1.875 *P* = 0.07), but there was a trend toward improvement. **(B)** EQ-VAS score significantly improved from the intake survey to the 6-week follow-up survey (Wilcoxon test *P* < 0.001), (***P < 0.001, ^+^*P* = 0.07).

Fifteen percent of patients reported that anxiety was the primary medical condition for which they were seeking medical cannabis treatment (Table 4). All patients who reported anxiety as their primary medical condition completed the DASS-21 and the EQL-5D-5L. There were no significant changes in the anxiety sub-scale of the DASS-21 (*t*_32_ = 1.875 *P* = 0.07, Figure 3A) after 6 weeks of medical cannabis treatment, though there was a trend toward improvement. For QOL, the EQ-VAS score significantly improved from the intake survey to the 6-week follow-up survey (Wilcoxon test *P* < 0.001, Figure 3B), indicating that overall QOL improved after 6 weeks of medical cannabis treatment for patients with anxiety.

Patients also provided self-reported changes in their anxiety after 6 weeks of medical cannabis treatment, with 85% reporting some improvement in their anxiety (Table 6).

**Table 6:** The number (and percentages) of the self-reported changes from patients with one of the top four medical conditions after 6 weeks of medical cannabis treatment.

**Medical condition**	**Change in medical condition** ***n*** **(%)**		
	**Deterioration**	**No change**	**Improvement**
Anxiety	3 (9.1)	2 (6.1)	28 (84.8)
PTSD	6 (18.8)	0 (0.0)	26 (81.3)
Arthritis and other rheumatic disorders	5 (19.2)	7 (26.9)	16 (61.5)
Sleep disorder	1 (3.7)	1 (3.7)	25 (92.6)

In the 6-week follow-up survey, patients who rated cannabis as helpful for treating their anxiety were also asked to indicate which cannabis product (if any) had been the most helpful. Thirty-two percent of patients selected high CBD products and 28% selected high THC products for treatment of anxiety (Table 7 and Supplementary Table 1).

**Table 7:** Information on preferred cannabis chemotypes for each of the top medical conditions reported by patients after 6 weeks of medical cannabis treatment.

**Medical condition**	**Cannabis chemotype**	***n* (%)**
Overall (number of respondents: 142)	High THC	46 (32.4)
	High CBD	44 (31.0)
	Balanced (THC:CBD)	18 (12.7)
	Other	32 (22.5)
	None	2 (1.4)
Anxiety (number of respondents: 25)	High THC	7 (28.0)
	High CBD	8 (32.0)
	Balanced (THC:CBD)	2 (8.0)
	Other	8 (32.0)
PTSD (number of respondents: 25)	High THC	10 (40.0)
	High CBD	3 (12.0)
	Balanced (THC:CBD)	5 (20.0)
	Other	7 (28.0)
Arthritis and other rheumatic disorders (number of respondents: 15)	High THC	3 (20.0)
	High CBD	8 (53.3)
	Balanced (THC:CBD)	3 (20.0)
	Other	1 (6.7)
Sleep disorder (number of respondents: 24)	High THC	9 (37.5)
	High CBD	6 (25.0)
	Balanced (THC:CBD)	4 (16.7)
	Other	5 (20.8)

*Only patients who self-reported improvements in their main medical condition (Table 5) were asked to provide preferred product information*.

#### Post-traumatic Stress Disorder

Fifteen percent of patients reported that PTSD was the primary medical condition for which they were seeking medical cannabis treatment (Table 4). Patients self-reported any changes they had experienced in their PTSD after 6 weeks of medical cannabis treatment, with 81% reporting some improvement in their PTSD (Table 6).

**Figure 4:**
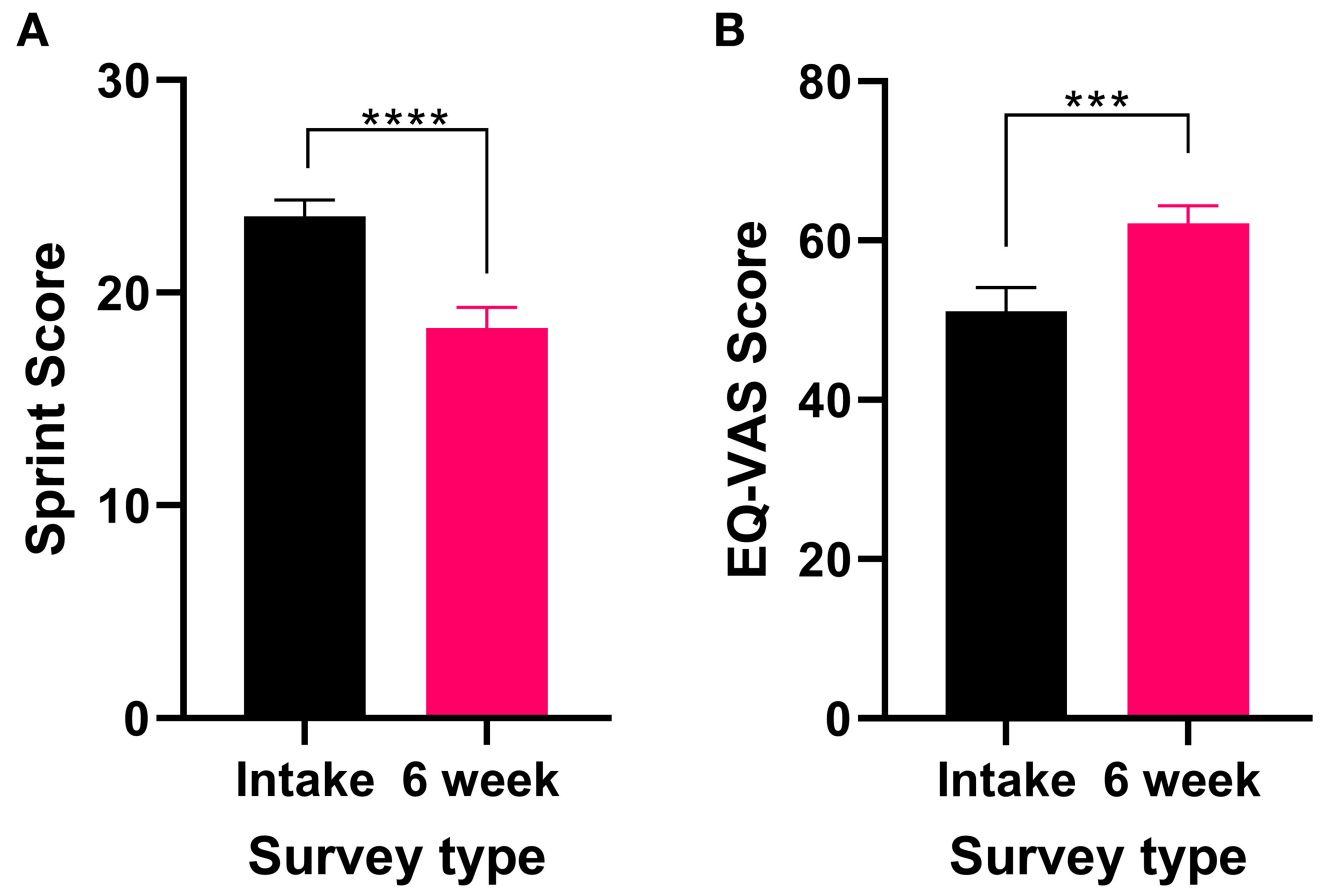
Validated survey scores from the intake survey (baseline) and 6-week follow-up survey for patients reporting PTSD as their primary medical condition. Overall, there was an improvement in patient's PTSD and QOL after 6 weeks of medical cannabis treatment. **(A)** SPRINT scores significantly decreased after 6 weeks of medical cannabis treatment (Wilcoxon test *P* < 0.0001). **(B)** EQ-VAS score significantly improved from the intake survey to the 6-week follow-up survey (*t*_31_ = 4.072 *P* < 0.001), (****P < 0.0001, ***P < 0.001).

All patients who reported PTSD as their primary medical condition completed the SPRINT and the EQL-5D-5L. There was a significant improvement in SPRINT scores (Wilcoxon test *P* < 0.0001) after 6 weeks of medical cannabis treatment (Figure 4A), indicating an improvement in PTSD symptoms from baseline to the 6-week follow-up.

In the 6-week follow-up survey version of the SPRINT, patients were asked “How much better do you feel since beginning medical cannabis treatment?” on a VAS scale. On average, patients reported feeling 49% (48.9% ± 18.2, range 10–75%) better since beginning medical cannabis treatment. For QOL, the EQ-VAS score significantly improved (*t*_31_ = 4.072 *P* < 0.001, Figure 4B) from the intake survey to the 6-week follow-up, indicating an overall improvement in QOL after 6 weeks of medical cannabis treatment for patients with PTSD.

Finally, in the 6-week follow-up survey, patients were asked to indicate which cannabis product (if any) had been the most helpful in treating their PTSD. Of those who rated cannabis as helpful for treating their PTSD, 40% selected high THC products (Table 7 and Supplementary Table 1).

#### Arthritis and Other Rheumatic Disorders

Thirteen percent of patients reported that arthritis or another rheumatic disorder was the primary medical condition for which they were seeking medical cannabis treatment (Table 4). Patients self-reported any changes they had experienced in their arthritis or other rheumatic disorders after 6 weeks of medical cannabis treatment, with 62% reporting some improvement (Table 6).

**Figure 5:**
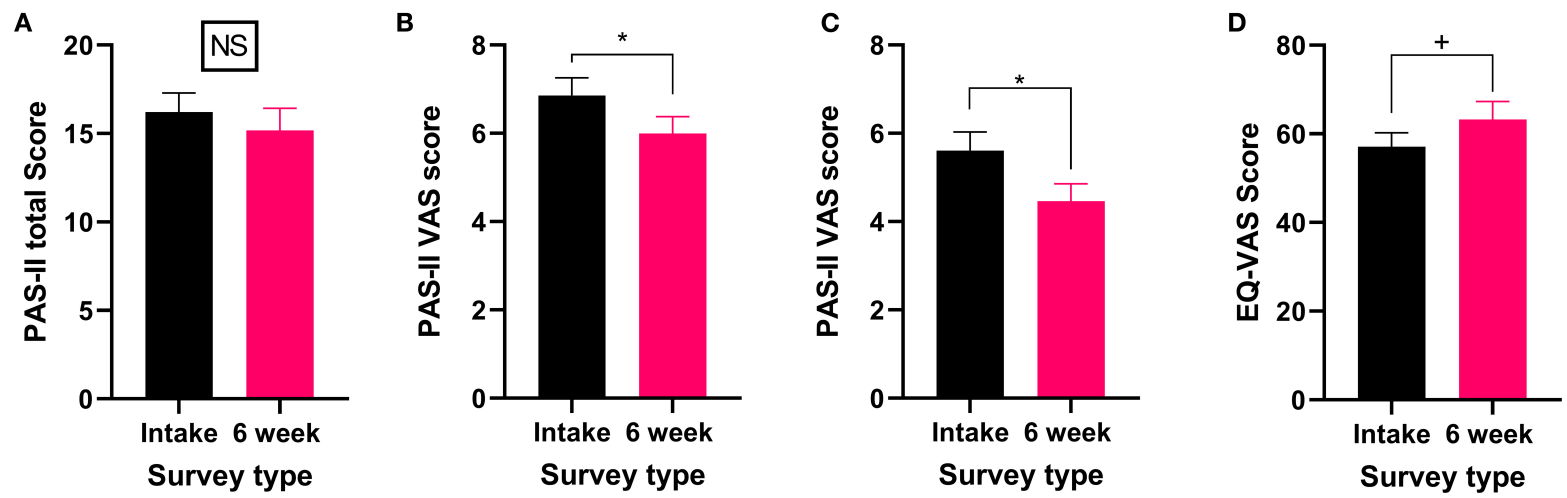
Validated survey scores from the intake survey (baseline) and 6-week follow-up survey for patients reporting arthritis or another rheumatic disorder as their primary medical condition. Overall while total PAS-II score did not improve, both the pain VAS and global activity VAS scores showed improvements after 6 weeks of medical cannabis treatment. **(A)** Total PAS-II score (Wilcoxon test *P* > 0.05) did not significantly change after 6 weeks of medical cannabis treatment. **(B)** The pain VAS score contained within the PAS-II (Wilcoxon test *P* < 0.05) significantly improved from the intake survey to 6-week follow-up survey. **(C)** The global activity VAS score contained within the PAS-II (Wilcoxon test *P* < 0.05) significantly improved after 6 weeks of medical cannabis treatment. **(D)** EQ-VAS score was not significantly altered from the intake survey to the 6-week follow-up survey (Wilcoxon test *P* = 0.09), though there was a small trend toward improvement. (*P < 0.05, +*P* = 0.09, NS *P* > 0.05).

All patients who reported experiencing arthritis or another rheumatic disorder completed the PAS-II and the EQL-5D-5L. While the total PAS-II score did not significantly change (Wilcoxon test *P* > 0.05, Figure 5A) after 6 weeks of medical cannabis treatment, both the pain VAS score (Wilcoxon test *P* < 0.05, Figure 5B) and the global activity VAS score (Wilcoxon test *P* < 0.05, Figure 5C) contained within the PAS-II significantly improved. Thus, patients with arthritis or another rheumatic disorder reported improvements specifically in pain and global activity after 6 weeks of cannabis treatment but not in their overall condition. For QOL, the EQ-VAS score was not significantly changed (Wilcoxon test *P* = 0.09, Figure 5D) from the intake survey to the 6-week follow-up survey, though there was a small trend toward improvement.

Finally, in the 6-week follow-up survey, patients were asked to indicate which cannabis product (if any) had been the most helpful in treating their arthritis or other rheumatic disorders. Of those who rated cannabis as helpful, 53% selected high CBD (Table 7 and Supplementary Table 1).

#### Sleep Disorder (Including Restless Leg Syndrome)

**Figure 6:**
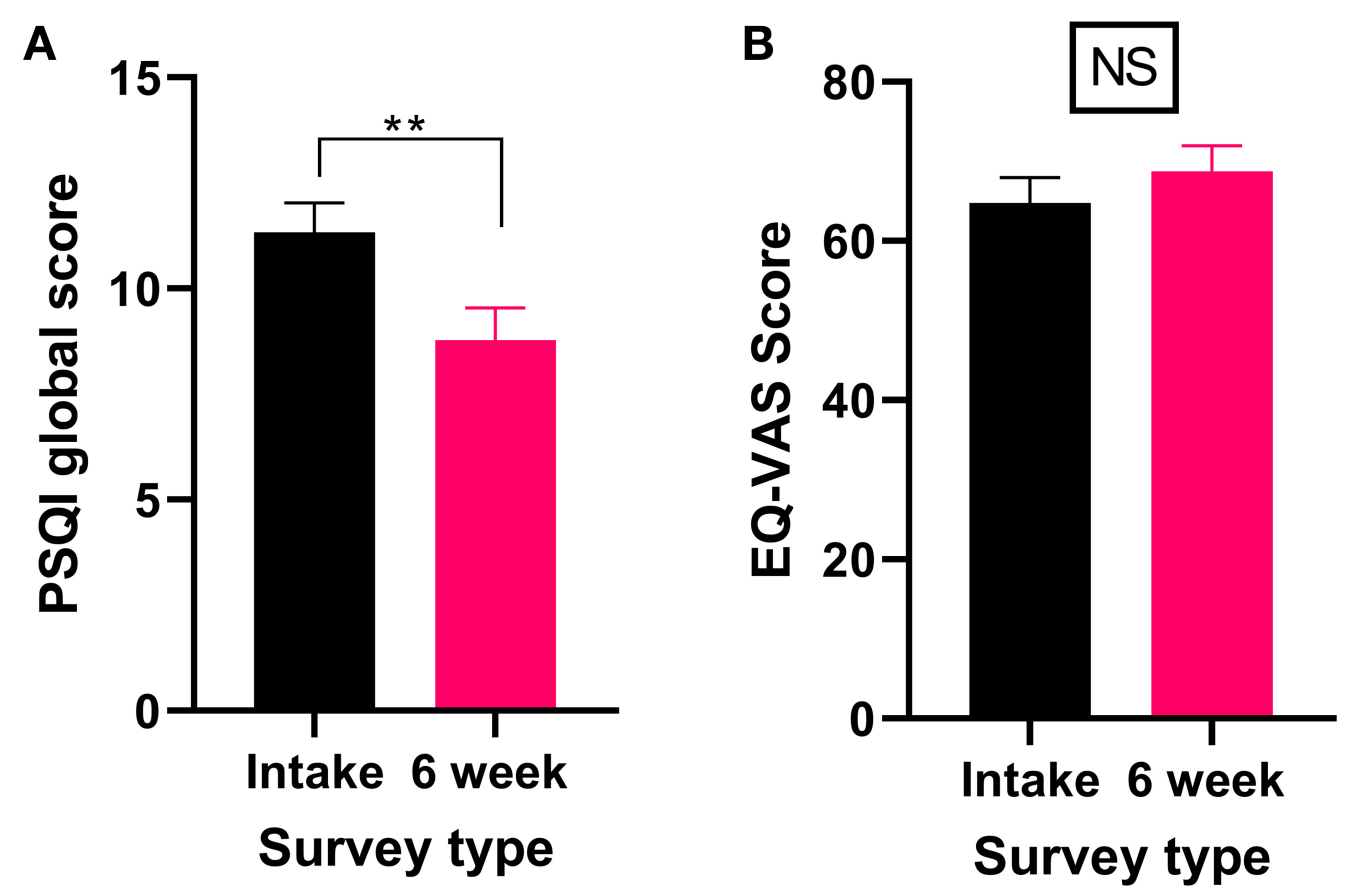
Validated survey scores from the intake survey (baseline) and 6-week follow-up survey for patients reporting a sleep disorder (including RLS) as their primary medical condition. Overall, there were significant improvements in the PSQI scores after 6 weeks of medical cannabis treatment, but no significant change was found in the QOL score. **(A)** PSQI scores significantly improved after 6 weeks of medical cannabis treatment (Wilcoxon test *P* < 0.001). **(B)** EQ-VAS score did not significantly change from the intake survey to the 6-week follow-up survey (Wilcoxon test *P* > 0.05), (**P < 0.01, NS *P* > 0.05).

Thirteen percent of patients reported that a sleep disorder was the primary medical condition for which they were seeking medical cannabis treatment (Table 4). All patients who reported a sleep disorder as their primary medical condition completed the PSQI and the EQL-5D-5L. There was a significant improvement in PSQI scores (Wilcoxon test *P* < 0.01) after 6 weeks of medical cannabis treatment (Figure 6A), indicating an improvement in sleep from baseline to the 6-week follow-up. For QOL, there were no significant changes in the EQ-VAS score (Wilcoxon test *P* > 0.05, Figure 6B) after 6 weeks of medial cannabis treatment.

Patients also provided self-reported changes in their sleep disorder after 6 weeks of medical cannabis treatment, with 93% reporting some improvement in their sleep disorder (Table 6).

In the 6-week follow-up survey, patients who rated cannabis as helpful for treating their sleep disorder were asked to indicate which cannabis product (if any) had been the most helpful. Thirty-eight percent selected high THC products as helpful for treating their sleep disorder (Table 7 and Supplementary Table 1).

### Side Effects

Patients were asked, “Have you experienced side effects during your medical cannabis treatment?” If patients reported experiencing side effects, they were asked to select “Which side effect have you experienced?” from a list of 17 options. Twenty percent of patients reported experiencing a total of 124 side effects after 6 weeks of medical cannabis treatment. The four most frequently reported side effects were dry mouth (22%), sleepiness (15%), restlessness (7%), and decreased memory (7%) (Table 8).

**Table 8:** Information on side effects experienced by patients after 6 weeks of medical cannabis treatment.

**Side effects**	***n* (%)**
**Experience side effects**	
No	171 (79.9)
Yes	43 (20.1)
**Top reported side effects**	(Total side effects reported *n* = 124)
1. Dry mouth	27 (21.8)
2. Sleepiness	18 (14.5)
3. Restlessness	9 (7.3)
3. Decreased memory	9 (7.3)

## Discussion

With increased legal access to medical cannabis and changing opinions, both socially and medically, patients are seeking cannabis to treat a broad range of medical conditions. However, while anecdotal evidence is abundant, healthcare practitioners routinely state they require further scientific evidence to become comfortable with authorizing cannabis. This study aimed to address the need for efficacy and safety data by surveying newly registered medical cannabis patients who completed a voluntary self-report survey before beginning medical cannabis treatment with a singular licensed producer (intake or baseline) and after 6 weeks of treatment (6-week follow-up).

Additionally, patients were asked what cannabis product they felt was most useful in treating their pain and/or specific main medical condition, with the aim of identifying which cannabis chemotype is most likely to help treat specific medical conditions (16). The majority of previous clinical studies have focused on investigating either a high THC, high CBD or balanced THC: CBD product, rather than exploring all three options and identifying which is most effective per medical condition. For example, the scientific evidence for anxiety treatment is primarily derived from the use of investigating high CBD products (26–31), whereas for pain, the majority of data has been generated from either high THC products [synthetic THC (32–34) or high THC herbal cannabis (35–37)] or nabiximols (a balanced THC:CBD product) (15, 38–41). There is also a disparity between the cannabinoid ratios used in pre-clinical and clinical research that requires further data to address. For example, PTSD pre-clinical studies have focused on the effects of CBD on PTSD symptoms (42–48) while clinical studies have reported the efficacy of high THC products or simply “cannabis” in treating PTSD (35, 49–51).

To our knowledge, this cohort of newly registered medical cannabis patients is one of few, if not the first, to be studied during a time period when both medical and adult-use cannabis was legal in Canada. This removes the potential influence of non-medical cannabis consumers in our patient cohort, providing a clearer representation of a patient cohort rather than a potential mixture of both adult-use consumers and medical patients. This is noteworthy as it has been a limitation of past observational studies carried out prior to the legalization of adult-use cannabis in Canada and in other jurisdictions where only medical cannabis is legal.

Our study found that patients report medical cannabis helps them to feel better overall and significantly improves several medical conditions and QOL (Table 9), findings which are supported by previously published research (52–57).

**Table 9:** Summary of results from evaluated validated questionnaires for each of the top four medical conditions after 6 weeks of medical cannabis treatment.

**Medical condition**	***n* (%)**	**Validated measure**	**Improvement in validated measure**	Improvement in QOL^*^
Recurrent pain	183 (85.5)	POQ-SF	*P* < 0.0001	*P* < 0.001
		Pain NRS	*P* < 0.0001	
Anxiety	33 (15.4)	DASS-21 anxiety sub-scale	*P* = 0.07	*P* < 0.001
PTSD	32 (15.0)	SPRINT	*P* < 0.0001	*P* < 0.001
Arthritis and other rheumatic disorders	28 (13.1)	PAS-II	*P* > 0.05	*P* = 0.09
		Pain VAS	*P* < 0.05	
		Global activity VAS	*P* < 0.05	
Sleep disorder	27 (12.6)	PSQI	*P* < 0.001	*P* > 0.05

* *As measured but the EQ-5D-5L sub-scale EQ-VAS*.

Our cohort consisted of 214 newly registered medical cannabis patients who had a mean age of 50.7 years, and 58% were male. Our population is comparable to other studies reporting that most medical cannabis patients are male (52, 56, 58–61). This predominantly male population contrasts with the general Canadian population, which shows closer to a 50:50 ratio between the sexes, as reported in the Canadian 2016 census (62). Additionally, this medical cannabis patient cohort consisted of mainly older patients, with 57% of them over 50 years of age. This supports previous findings by Eurich et al., who analyzed patient data collected from the Canadian Cannabis Clinics between 2014 and 2016 (58). It is interesting to note that patient age and sex distribution is not significantly different from the cohort Eurich et al. reported on, though our study took place when both adult-use and medical cannabis were legal in Canada while only medical cannabis was legal during Eurich et al.'s study (58). Thus, we propose the legalization of adult-use cannabis has not significantly altered the demographics (in terms of age and sex) of Canadians that seek out medical cannabis treatment.

Additionally, as others have reported (52, 54–56), we found that over half of the newly registered medical cannabis patients had previous experience with cannabis before registering to use medical cannabis with the licensed producer, MedReleaf^®^. This experience with cannabis, and continuation to seek out cannabis as a therapy, adds support to our findings that patients feel cannabis is efficacious in treating their medical conditions as one can hypothesize that in the absence of effects, patients would discontinue their cannabis treatment. It also aligns with the manageable side effects that our patients reported, (with only 20% of patients reporting side effects, such as dry mouth and sleepiness), as it is logical to conclude that if patients were experiencing serious adverse events, they would be unlikely to continue their medical cannabis therapies.

### Recurrent Pain

Eighty-six percent of our medical cannabis patient cohort reported they experience recurrent pain, which is consistent with past research finding that patients primarily seek out medical cannabis to treat chronic pain (36, 37, 58). Here, we show that our patients had significant pain improvements, as measured by the POQ-SF, a validated survey for pain, after 6 weeks of medical cannabis treatment. In addition to pain improvements over the six-week treatment period, our patients had improved QOL as measured by the EQ-5D-5L. Our results provide real-world evidence for the benefits of medical cannabis in the treatment of recurrent pain. Additionally, with the growing body of evidence for the possible role of cannabis in opioid-sparing (63–65), our findings that both pain and QOL improve with only 6 weeks of medical cannabis treatment highlights the importance of future clinical studies that explore the relationships and mechanism(s) behind cannabis and pain as a possible means to reduce the negative impacts of chronic opioid use (66).

An additional aim of this study was to identify which types of chronic pain (i.e., neuropathic or nociceptive) our recurrent pain patients were suffering from and if the type of pain impacted whether the patients reported benefits from cannabis treatment. Thirty-one percent of our recurrent pain patients reported they had nociceptive pain, while 22% self-reported neuropathic pain. Interestingly, both pain type groups showed improvements in pain as measured by the POQ-SF after 6 weeks of cannabis treatment. This improvement in both nociceptive and neuropathic pain is in contrast to a real-world evidence study by Ueberell et al. that found 12-weeks of nabiximols treatment significantly improved pain only for patients with chronic neuropathic pain and mixed pain while it was not effective and/or worsened pain symptoms for patients with nociceptive pain (15). This difference in findings may be due to the different THC:CBD ratios that our medical cannabis patients had available to them. For instance, our patients with recurrent pain reported that they found high CBD products (33%) and high THC products (31%) to be effective in treating their pain symptoms whereas, Ueberall et al.'s patients were only prescribed nabiximols, a balanced THC:CBD product (15). Our findings that 33% of patients with recurrent pain found high CBD products helpful in treating their pain is novel as to date, the majority of studies investigating the possible relationship between medical cannabis and pain have focused on high THC products (32–34, 36, 37, 66) or balanced THC:CBD products, like nabiximols (15, 38–41). While there is a significant body of pre-clinical work that has examined CBD's role as an anti-inflammatory agent and analgesic (67–74), this has not meaningfully translated into clinical investigation yet.

Overall, our findings show that medical cannabis treatment over 6 weeks provides benefits to patients with recurrent pain, both by significantly reducing pain as well as improving QOL. We propose that future clinical studies focus on the efficacy of cannabis in treating chronic pain specifically assess a range of THC:CBD ratios against the particular type of pain their patients experience. Until which time further studies are conducted, healthcare practitioners may want to explore different THC:CBD ratios when treating chronic pain patients in order to determine what product is most efficacious for their patients.

### Main Medical Condition

Similar to other cannabis-focused survey publications, both in Canada (58, 75) and internationally (53, 61, 76, 77), our cohort of medical cannabis patients reported a diverse range of medical conditions, with pain, mental health conditions, sleep disorders and rheumatic disorders being the most commonly reported. While 36% of patients reported they felt that there were definite and real improvements in their overall health, more impressive is that for the top four medical conditions (anxiety, PTSD, arthritis and other rheumatic disorders, and sleep disorders), over 60% of patients self-reported improvements for each of these medical conditions. This contrasts with findings from other publications and the currently available conclusions made by large reviews on the efficacy of cannabinoids for many of these conditions (78, 79). In addition to providing self-reported improvements in overall health and main medical conditions, patients completed both condition specific and QOL validated surveys to assess what effect 6 weeks of medical cannabis treatment had on their main medical condition.

While 60% of our medical cannabis cohort reported improvements in their medical conditions, findings that align with previously published studies (52, 54–56), not all of the medical conditions examined in our study showed improvement. We report that patients who stated anxiety or arthritis and other rheumatic disorders as their main medical condition for seeking out cannabis treatment did not experience significant improvement in all aspects of their respective conditions after 6 weeks of cannabis treatment (Table 9).

#### Post-Traumatic Stress Disorder

Previous research employing self-reported surveys have shown that medical cannabis patients with PTSD report significant reductions in their PTSD-specific symptoms including pain severity (80, 81), the impact of PTSD on social and family life (80), general mood (82), sleep (81, 82), concentration (82) and overall QOL (82). Additionally, when patients self-reported their PTSD-related symptoms immediately after using medical cannabis, all symptoms were reduced by more than 50% (50). Specifically, intrusive thoughts, flashbacks, anxiety and irritability were all found to be decreased (50). The data presented here supports these previous findings as we show that after 6 weeks of medical cannabis treatment, our PTSD patients reported feeling 49% better and had improvements in their PTSD, as measured by the SPRINT. In addition to these changes in validated measures, 81% of our PTSD patient cohort also self-reported improvements in their PTSD after medical cannabis treatment and a significant improvement in QOL was identified. Thus, 6 weeks of medical cannabis treatment significantly benefited both overall QOL and PTSD symptoms in our patients with PTSD.

Of our patients who self-identified as having PTSD, 40% reported that high THC products were most helpful for treating their PTSD. While the research is limited, our findings of a preference for high THC products agree with previously self-reported data (35, 81). To date, primarily synthetic THC (man-made chemical compounds rather than THC from cannabis plants) has been investigated as a possible therapy for PTSD. These studies have shown that synthetic THC can significantly reduce nightmares and enhance general well-being in patients with PTSD (83). However, here we provide evidence that the combination of phytocannabinoids and/or terpenes, specifically cannabis products high in THC, are reported by patients to effectively relieve their PTSD and provide measurable improvements in PTSD via the SPRINT.

From the benefits seen in patients with PTSD and the currently available scientific literature, it appears that cannabis may help relieve PTSD symptoms (50, 80–82, 84, 85). While these results are positive, it should be noted that some research studies have shown negative impacts of cannabis use in patients with PTSD as well (49, 51). Thus, more research is needed to examine how effective cannabis-based products are at treating PTSD (86) and if there is a specific ratio of THC:CBD that would be more efficacious than others in relieving PTSD symptoms.

#### Sleep Disorders (Including RLS)

A benefit commonly associated with cannabis is improved sleep, with many people routinely using cannabis as a sleep aid (61, 87, 88). Currently, robust, interventional clinical trials examining the efficacy of medical cannabis in aiding sleep are limited. Most of the information on the relationship between cannabis and sleep comes from studies examining sleep changes as a secondary outcome rather than a primary objective (89). While limited, the research to date does suggest that cannabinoids could improve sleep quality and decrease both sleep disturbances and sleep onset latency (35, 79, 89). In line with these findings, we report significant improvements in sleep, as measured by the PSQI, in patients who stated they were seeking medical cannabis to treat sleep disorders (including RLS) after 6 weeks of medical cannabis treatment. Additionally, 93% of these patients self-reported improvements in their overall condition. While we found improvements in sleep, this cohort showed no overall change in QOL. This may be due to the fact that sleep issues are often part of another undiagnosed health condition. Thus, while we saw improvements in sleep in our patient cohort looking to treat sleep disorders, the cannabis treatment may have only addressed one aspect (i.e., sleep issues) of a larger health issue and so QOL was unchanged.

Of the patients who self-identified as having sleep disorders, patients reported that high THC (38%) products were most helpful for treating their sleep disorders. Overall, these findings of preference for high THC products agree with previous research that showed THC improves subjective sleep quality and decreases sleep disturbances (89). It is worth noting that to date, most of the research on the relationship between THC and sleep has utilized synthetic THC rather than THC derived from cannabis plants. Here we report that patients with sleep issues find the combination of phytocannabinoids and/or terpenes, specifically cannabis products high in THC, to be effective.

#### Arthritis and Other Rheumatic Disorders

The role medical cannabis may play in treating arthritis and other rheumatic disorders is complex, and the research to date has been limited. Several pre-clinical studies have looked at CBD's role in the treatment of arthritis, focusing on pain and inflammation, finding positive results (72, 90, 91). In agreement with this pre-clinical research, 53% of our patients with arthritis and other rheumatic disorders reported that high CBD products were most helpful for treating their condition. However, while patients felt these products improved their condition, this did not translate to a significant improvement in their overall PAS-II score. Nonetheless, both the pain and global activity sub-scores of the PAS-II did significantly improve after 6 weeks of cannabis treatment. These latter findings are supported by a previous study by Blake et al. which found that nabiximols effectively reduced pain and improved sleep quality in patients with rheumatoid arthritis (92). Unfortunately, Blake et al. did not look at improvements in overall QOL or in arthritis specific measures and so we are unable to draw further comparisons around overall condition improvement (92). However, in our study, the lack of change in overall PAS-II scores is further reflected in the unchanged QOL scores. It is possible that with extended periods (>6 weeks) of treatment with medical cannabis there may be changes in QOL and overall PAS-II scores for patients with arthritis and other rheumatic disorders, but this remains to be investigated. Nevertheless, the improvements in both pain and global activity experienced by these patients, coupled with the fact that 62% did self-report improvements in their arthritis and other rheumatic disorders, emphasizes the need for future research into the role cannabis may play in treating arthritis and other rheumatic disorders.

#### Anxiety

Of those patients who self-identified as having anxiety, 85% self-reported finding improvements in their anxiety after 6 weeks of medical cannabis treatment. This is in agreement with other self-reported studies that report improvements in anxiety with medical cannabis (35, 93). While the results presented here indicate self-reported improvements in anxiety, we did not find any significant changes in anxiety as measured by the DASS-21 anxiety sub-scale. This is somewhat surprising as there is a growing body of scientific research that indicates that cannabis, mainly CBD, may be helpful in treating anxiety symptoms (26–31). However, there are a few differences between this patient cohort with anxiety and those from the published literature that may explain the disparity between our findings and the literature. Firstly, the patients in this study may have had a variety of different types of anxiety, including solely self-diagnosed rather than physician diagnosed, and as many of the published studies on anxiety have been in non-anxious populations experiencing events that may cause anxiety like feelings (26–28, 94), the findings from these studies may not directly relate to our patient population. Secondly, while this anxiety patient cohort showed no significant improvements in anxiety directly, there were improvements in their QOL. This is not necessarily surprising, as overall well-being and specific symptoms have multiple causes and can be affected by several factors. Thus, while the anxiety itself did not necessarily improve, the use of medical cannabis for 6 weeks may have alleviated other underlying symptoms, leading to an overall improvement in QOL.

Another interesting finding from the patients who self-identified as having anxiety is that our patients reported that both high CBD (32%) and high THC (28%) products were most helpful for treating their anxiety. This almost equal split between the proportion of patients finding anxiety relief with high THC and high CBD products seems to contrast the current research focus on the use of CBD to treat anxiety (26–31); previous studies have shown most patients report using CBD to treat their anxiety and that CBD treatment is found to improve anxiety (95, 96). While this at first appears to conflict with the common belief that only CBD would be useful for treating anxiety, a recent review of the clinical research in anxiety and depression found that THC improved anxiety in individuals with other medical conditions, where anxiety was not the primary diagnosis (97). Thus, this may be the case for some of our patient cohort where their anxiety is a co-morbidity with another, or many other, medical condition(s). For instance, anxiety is a common co-morbidity of chronic pain (58) and 86% of our patients reported they experienced recurrent pain, the latter perhaps improving with cannabis treatment leading to an overall better QOL score at the 6 week follow up though their anxiety remained unchanged. This hypothesis also supports our earlier point above, that patients who self-reported they had anxiety may have other underlying conditions where medical cannabis treatment provided improvements, leading to benefits to overall QOL but not necessarily any change in their anxiety symptoms. Additionally, while evidence for the use of THC in the treatment of anxiety is minimal, other self-reported surveys in medical cannabis patients have found that high THC products are most associated with condition improvement (35). This indicates that THC may play a more complex role in relation to anxiety. Therefore, we would suggest future clinical studies examining cannabis as a means to treat anxiety include multiple THC:CBD ratios in order to identify the optimal ratio to treat this condition.

## Conclusion

Aligning with previously published studies (52, 54–56), we report that over 60% of the medical cannabis cohort reported improvements in their medical conditions. Thus, we conclude that in general, this real-world data shows that a large proportion of medical cannabis patients report moderate to substantial benefits from cannabis, both in terms of their overall condition and general well-being. While these results are promising, cannabis treatment was not a remedy for all, as our findings show that medical cannabis did not lead to significant improvements in all conditions we examined.

Our results show significant improvements in recurrent pain, PTSD, and sleep disorders (including RLS) after 6 weeks of medical cannabis treatment using validated surveys. Our findings from patients who reported arthritis and other rheumatic disorders are complex. While we report that there were improvements in pain and global activity sub-scores, we did not see overall changes in validated survey scores. We also report that patients who stated anxiety as their main medical condition did not experience significant changes in their anxiety after 6 weeks of cannabis treatment, though there were QOL improvements. These QOL improvements may show an alleviation of other underlying symptoms, leading to improved QOL, but not changes in anxiety specifically.

Some limitations of our study are as follows. Firstly, our retention rate from the baseline survey to the 6-week follow-up survey was approximately 35%. This is lower than we had anticipated and is an aspect we aim to address in future iterations of the patient survey by decreasing the time it takes to complete the survey and providing a small incentive for completing the surveys to increase the number of patients per medical condition. Secondly, this sample might not be generalizable to the entire medical cannabis patient population in Canada because patients self-selected to be in the study, we did not collect information on patients who refused the study, and all patients were from a single licensed producer. Additionally, we were unable to meaningfully collect information about dosing and frequency of dosing. This is information we know healthcare practitioners need and an item we aim to collect in the future. This data may also help us tease out why in some medical conditions, like anxiety, we had patients reporting both high THC and high CBD products as effective in treating their conditions. It is possible that a large dose of a high THC cannabis product could provide similar levels of CBD concentrations to the consumption of a small dose of a high CBD product, and vice versa. And lastly, as we noted above, it is possible we may have seen greater effects if we followed our patients over a longer period. However, while 6 weeks is a shorter timeline, we would contest that in most cases, if a patient does not experience meaningful improvements within 6 weeks, they are unlikely to continue with a treatment long term.

While randomized controlled trials (RCT) are considered the gold standard in terms of generating scientific evidence on pharmaceuticals, the utilization of cannabis as medicine is legislatively unique as a therapeutic intervention, due to its widespread use and legal status in many countries around the world. Because of this, the gathering of real-world data via surveys such as ours are imperative as a means to gather patient-reported safety and efficacy in a low cost and timely manner to support safe and effective medical cannabis authorizations in jurisdictions already doing so. Overall, we have provided new real-world evidence to support the use of medical cannabis in a number of different medical conditions as a means to immediately provide the scientific evidence healthcare practitioners routinely state they require and to inform the future clinical studies needed to generate efficacy and safety data that will ultimately support the drafting of future regulatory guidelines surrounding medical cannabis use.

## Data Availability Statement

The original contributions presented in the study are included in the article/Supplementary Material, further inquiries can be directed to the corresponding author/s.

## Ethics Statement

Ethical review and approval was not required for the study on human participants in accordance with the local legislation and institutional requirements. The patients/participants provided their written informed consent to participate in this study.

## Author Contributions

SC performed all data and statistical analysis. SC and SEL wrote the manuscript. PD created the survey on the Qualtrics platform and selected validated survey tools for each of the specified medical conditions. JP contributed to the writing of the manuscript. All authors have made substantial contributions to the conception and design of the study or drafting of the manuscript.

## Conflict of Interest

All authors work for Aurora Cannabis Inc.^®^ which is a for-profit company licensed for the cultivation and sale of medical cannabis.
