# Supplementary material for: Evaluation of Patient Reported Safety and Efficacy of Cannabis From a Survey of Medical Cannabis Patients in Canada
Source: Front Public Health. 2021 May 20;9:626853. doi: 10.3389/fpubh.2021.626853 (PMC8172603; doi:10.3389/fpubh.2021.626853)
Supplement: Supplementary file 1 [file Data_Sheet_1.docx]

Supplementary Material

# Supplementary Figures and Tables

## Supplementary Tables

**Supplemental Table 1.** Information on the top cannabis cultivars and product types for recurrent pain and for each of the most common medical conditions reported by patients after six weeks of medical cannabis treatment.

| **Medical condition** | **Top cannabis**  **cultivars (chemotype)** | **Top cannabis product**  **type** |
| --- | --- | --- |
| Overall | \| Avidekel (High CBD) \| \| --- \| \| Sedamen (High THC) \| \| Midnight (Balanced THC:CBD) \| | \| Cannabis oil \| \| --- \| \| Cannabis softgel \| \| Dried herbal cannabis \| |
| Recurrent pain | \| Avidekel (High CBD) \| \| --- \| \| Sedamen (High THC) \| \| Midnight (Balanced THC:CBD) \| | \| Cannabis softgel \| \| --- \| \| Cannabis oil \| \| Dried herbal cannabis \| |
| Anxiety | \| Avidekel (High CBD) \| \| --- \| \| Sedamen (High THC) \| | \| Cannabis softgel \| \| --- \| \| Cannabis oil \| \| Cannabis vaporizer \| |
| PTSD | \| Eran Almog (High THC) \| \| --- \| \| Midnight (Balanced THC:CBD) \| \| Avidekel (High CBD) \| | \| Cannabis oil \| \| --- \| \| Dried herbal cannabis \| \| Cannabis softgel \| |
| Arthritis and other  rheumatic disorders | \| Avidekel (High CBD) \| \| --- \| \| Sedamen (High THC) \| \| Midnight (Balanced THC:CBD) \| | \| Cannabis oil \| \| --- \| \| Cannabis softgel \| |
| Sleep disorder | \| Avidekel (High CBD) \| \| --- \| \| Sedamen (High THC) \| \| Midnight (Balanced THC:CBD) \| | \| Cannabis oil \| \| --- \| \| Cannabis softgel \| \| Cannabis vaporizer \| |

## Supplementary Figures

**
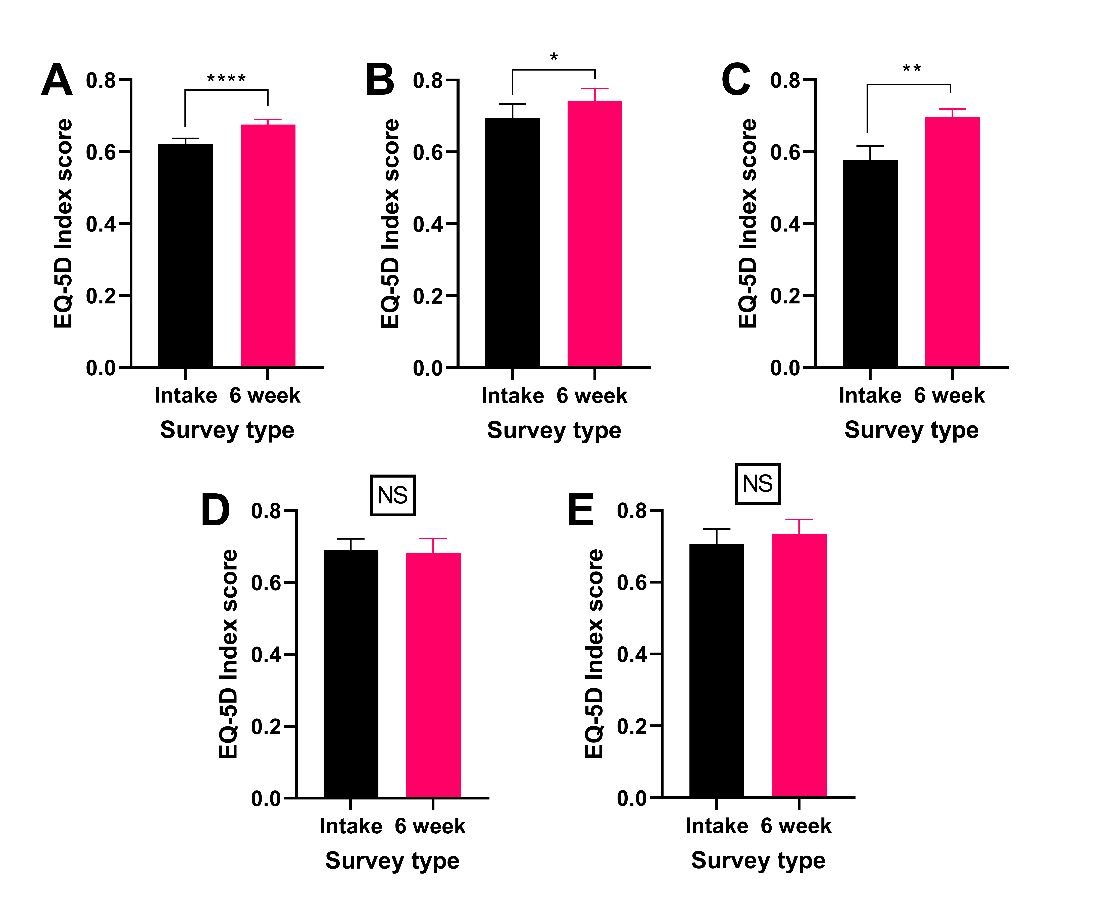
**

**Supplemental Figure 1:** EQ-5D index scores from the intake (baseline) and 6-week follow-up surveys. **(A)** EQ-5D index scores for patients with recurrent pain showed significant improvement after 6-weeks of medical cannabis treatment (Wilcoxon test *P*<0.0001), **(B)** EQ-5D index scores for patients with anxiety showed significant improvement after 6-weeks of medical cannabis treatment (t_32_=2.499, *P*<0.05), **(C)** EQ-5D index scores for patients with PTSD showed significant improvement after 6-weeks of medical cannabis treatment (Wilcoxon test *P*<0.01), **(D)** EQ-5D index scores for patients with arthritis and other rheumatic disorders showed no change after 6-weeks of medical cannabis treatment (Wilcoxon test *P*>0.05), **(E)** EQ-5D index scores for patients reporting sleep disorders showed no change after 6-weeks of medical cannabis treatment (Wilcoxon test *P*>0.05) (*****P*< 0.0001, ***P*< 0.01, **P*< 0.05, NS *P*>0.05).

**
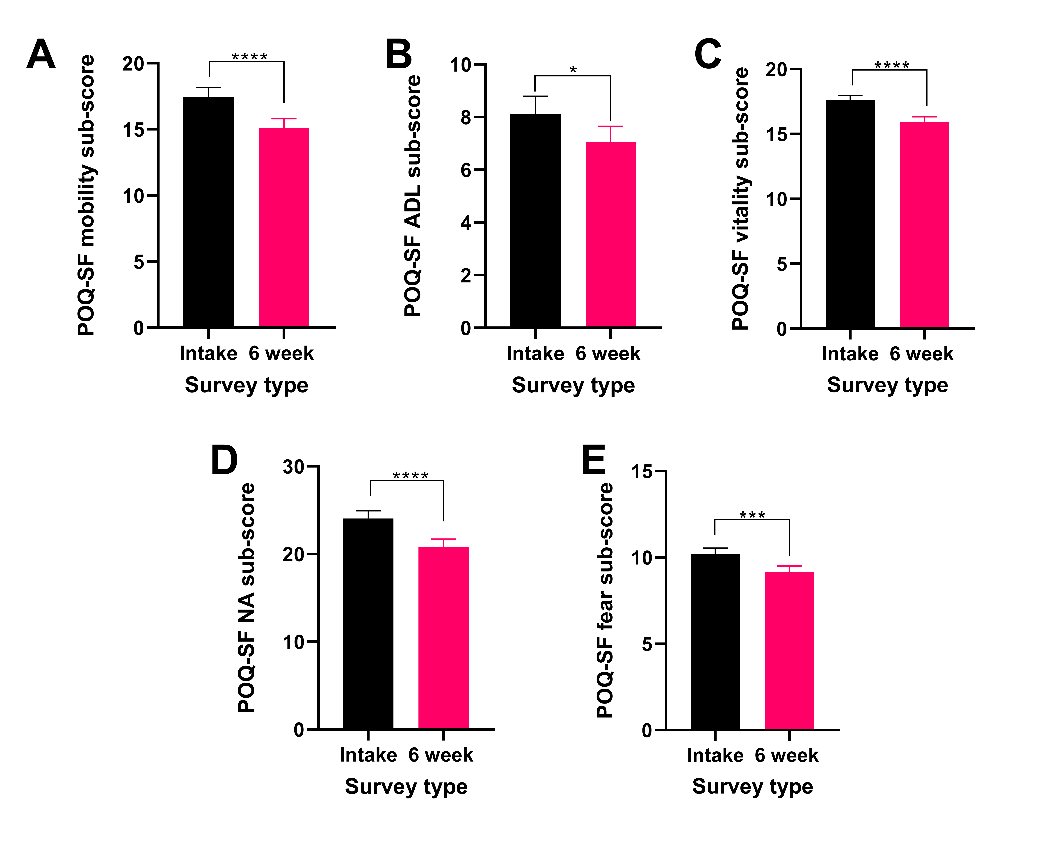
**

**Supplemental Figure 2:** Validated survey scores from the intake survey (baseline) and 6-week follow-up survey for patients reporting recurrent pain. All five subscales of the POQ-SF significantly improved after 6 weeks of medical cannabis treatment. **(A)** Mobility sub-score (t_182_=4.74 *P*<0.0001), **(B)** Activities of daily living (ADL, Wilcoxon test *P*<0.05), **(C)** Vitality (Wilcoxon test *P*<0.0001), **(D)** Negative affect (NA, t_182_=4.75 *P*<0.0001), **(E)** Fear (t_182_=3.66 *P*<0.001) (*****P*< 0.0001, ****P*< 0.001, **P*< 0.05).
